# The role of histogram analysis in diffusion-weighted imaging in the differential diagnosis of benign and malignant breast lesions

**DOI:** 10.1186/s12911-020-01257-0

**Published:** 2020-09-21

**Authors:** Ya-Nan Jin, Yan Zhang, Jing-Liang Cheng, Xiao-Pan Zhang, Ying Hu, Xiao-Ning Shao

**Affiliations:** Department of Magnetic Resonance Imaging, The First Affiliated Hospital of Zhengzhou University, No. 1 of Jianshe East Road, Erqi District, Zhengzhou, 450052 China

**Keywords:** Breast tumor, IVIM, Diffusion weighted imaging, Magnetic resonance imaging, Histogram metrics, Differential diagnosis

## Abstract

**Background:**

The present study aims to investigate the role of histogram analysis of intravoxel incoherent motion (IVIM) in the differential diagnosis of benign and malignant breast lesions.

**Methods:**

The magnetic resonance imaging and clinical data of 55 patients (63 lesions) were retrospectively analyzed. The multi-b-valued diffusion-weighted imaging image was processed using the MADC software to obtain the gray-scaled maps of apparent diffusion coefficient (ADC)-slow, ADC-fast and f. The MaZda software was used to extract the histogram metrics of these maps. Combined with the conventional sequence images, the region of interest (ROI) was manually drawn along the edge of the lesion at the maximum level of the gray-scale image, and the difference of the data was analyzed between the benign and malignant breast lesions.

**Results:**

There were 29 patients with 37 benign lesions, which included 23 fibroadenomas, 6 adenosis, 1 breast cysts, 4 intraductal papillomas, and 3 inflammations of breast. Furthermore, 26 malignant lesions in 26 patients, which included 20 non-specific invasive ductal carcinomas, 5 intraductal carcinomas and 1 patient with squamous cell carcinoma. The ADC-slow (mean and the 50th percentile) and f (minimum, mean, kurtosis, the 10th percentile and 50th percentile) of these malignant breast lesions were significantly lower than those of benign lesions (*P* < 0.05), while ADC-fast (kurtosis) and f (variance, skewness) of these malignant breast lesions were significantly higher than those of benign lesions (*P* < 0.05).

**Conclusion:**

The histogram analysis of ADC-slow (mean and the 50th percentile), ADC-fast (kurtosis) and f (minimum, mean, kurtosis, the 10th percentile and 50th percentile. Variance, skewness) can provide a more objective and accurate basis for the differential diagnosis of benign and malignant breast lesions.

## Background

The incidence of breast cancer is continuously increasing and tends to occur more in younger patients [1]. In China, the number of new cases of and deaths from breast cancer account for 12.2 and 9.6%, respectively, of cases in the world, and the incidence of breast cancer has increased twice as fast as that in the rest of the world. Furthermore, the average age of patients diagnosed with breast cancer in China is in the 45–55 age range, which is younger than that of Western women [2]. Therefore, the early detection and diagnosis of breast cancer are critical. Although punch biopsy remains the gold standard [3] for the diagnosis of breast cancer, some studies have shown that only 53.1% of patients are diagnosed with breast cancer by punch biopsy, indicating that there are some defects and missed diagnoses in the diagnosis of breast cancer [4]. Therefore, the significance of imaging in the diagnosis of breast cancer is growing. Heterogeneity is an important feature of malignant breast tumors that greatly affects the choice of treatment methods and the efficacy of chemotherapy drugs [5]. Due to its high soft-tissue resolution, magnetic resonance imaging (MRI) has increasingly been used in the screening and diagnosis of breast lesions [6]. Some studies have shown that the MRI results for breast cancer are consistent with pathological sections. Therefore, its sensitivity is very high. In addition, since MRI is free of radiation and can be performed repeatedly, it has become one of the safest and most effective diagnostic modalities for breast cancer [7]. However, there is some overlap in the examination of benign and malignant breast lesions by MRI. For example, breast cancer shows a high signal intensity on diffusion-weighted imaging (DWI), and some benign breast lesions also show a high signal intensity, which limits the clinical application of MRI [7].

In recent years, DWI, as a functional imaging method that can reflect tissue microenvironments, has been increasingly used in clinical diagnosis [8]. However, DWI based on intravoxel incoherent motion (IVIM) can more accurately describe the diffusion motion characteristics of water molecules in the tissue microenvironment, thereby more accurately reflecting the heterogeneity of breast lesions [8]. Gray-scale histogram analysis can quantify the signal intensity distribution in the lesions to more objectively reflect the tissue heterogeneity in the lesion [9]. Gray-scale histogram analysis is an intuitionistic texture analysis based on statistics. The commonly used first-order statistical parameters include the average, maximum, minimum, variance, skewness, and kurtosis of the signal strength in the pixel [9]. This can more objectively analyze the image information, and provide a more reliable basis for the identification, classification, efficacy, and prognosis evaluation of benign and malignant tumors. Using the gray-scale histogram of DWI may enhance the sensitivity of the MRI, thereby improving the diagnostic efficiency of MRI for breast cancer [9].

Based on the above assumptions, the present study retrospectively analyzed the MRI images and clinical data of 55 patients (63 lesions). The difference in comparative data in benign and malignant breast lesions was analyzed to investigate the role of the histogram analysis of DWI in the differential diagnosis of benign and malignant breast lesions.

## Methods

### Patients

The MRI images and clinical data of patients who met the below criteria in the First Affiliated Hospital of Zhengzhou University from December 2015 to December 2017 were collected and retrospectively analyzed. The study was conducted ethically in accordance with the World Medical Association Declaration of Helsinki. All subjects gave their written informed consent, and the study protocol was approved by the Ethics Committee of the First Affiliated Hospital of Zhengzhou University.

Patient criteria for inclusion: (1) patients who completed the T1-weighted imaging (T1WI), T2-weighted imaging (T2WI), T1WI dynamic enhancement scan and diffusion-weighted imaging (DWI); (2) within two weeks of the completion of the scan, an operation puncture or pathological puncture was required to confirm the pathological results; (3) no treatment was performed before the scan; (4) the scanning image was of good quality, without obvious motion artifacts. All 55 patients were female, and their ages ranged from 12 to 70 years, with a median age of 42 years.

### Equipment and methods

A GE Discovery 750 3.0 T superconducting magnetic resonance and an 8-channel breast-specific phase-controlled coil were used. The patient was placed in a prone position, and the feet went in first.

1. *MRI plain scan parameters*

- Axial T1WI: TR/TE 640 ms/7.6 ms; FOV 320 mm × 320 mm;
- matrix 512 × 512;
- layer thickness/interlayer spacing 4 mm/1 mm;
- axial fat inhibition.
- T2WI: TR/TE 2587 ms/85 ms;
- FOV 320 mm × 320 mm;
- matrix 512 × 512;
- layer thickness/interlayer spacing 4 mm/1 mm.
- DWI: b = 0, 20, 50, 100, 150, 200, 400, 800, 1200, 1600, 2000, 2500, 3000 and 4000 s/mm^2^;
- number of excitation (NEX) corresponding to 1, 1, 1, 1, 1, 2, 2, 2, 4, 4, 6, 6, 8 and 10;
- TR/TE 3600 ms/76 ms;
- FOV 320 mm × 320 mm, matrix 256 × 256; layer thickness/interlayer spacing 4 mm/1 mm.

(2) *Contrast-enhanced magnetic resonance imaging (CE-MRI)*

- Axial-position volume imaging sequence Vibrant: TR/TE 3.9 ms/1.7 ms;
- FOV 360 mm × 360 mm;
- matrix 512 × 512;
- layer thickness/interlayer spacing 1.4 mm/1.0 mm.

After the end of the mask scan, a high pressure syringe was used to inject the Gd-DTPA through the elbow vein at a flow rate of 2.0 ml/s and a dose of 0.1 mmol/kg.

After the injection, 20 ml of normal saline was added to flush the tube. The dynamic scan was started after delaying for 20 s. Then, five time phases were scanned, and each phase was performed for 59 s.

### Image analysis and data measurement

The multi-b-valued DWI image was processed using the MADC software to obtain the gray-scaled maps of ADC-slow, ADC-fast, and f. The lesion image information was extracted using the MaZda software. Combined with a conventional sequence image, the region of interest (ROI) was manually drawn along the edge of the lesion at the maximum level of the lesion on the gray-scale map. Based on the bi-exponential model of voxel incoherent motion theory, ADC is separated into ADC-slow which represents the real diffusion motion of water molecules and ADC-fast which represents the micro perfusion in tissues, while f represents the perfusion fraction. The multi-b-valued DWI image was transferred to GE ADW 4.5 post-processing workstation. The gray-scale map of ADC-slow, ADC-fast and f was obtained using the MADC software in the Functool toolkit for post-processing. All post-processing images and other sequence images of the patients were derived in. BMP format, in order to make the image window width and window level consistent. The informations of these lesion images were extracted using the MaZda software. Combined with the conventional sequence images, the maximum level of the lesion was selected on the gray-scaled map of ADC-slow, ADC-fast and f, and the region of interest (ROI) was manually sketched along the edge of the lesion. Before extracting the image data, all images were standardized at the gray-scale level, in order to reduce the impact of brightness and contrast changes on the results [1]. All gray-scale histograms of all ROIs were automatically generated by the software, and the gray minimum, maximum, mean, variance, skewness, kurtosis, 1st percentile, 10th percentile, 50th percentile, 90th percentile and 99th percentile were obtained.

### Statistical analysis

The SPSS 21.0 statistical software was used to analyze the data and compare the difference in data between benign and malignant breast lesions. Kolmogorov-Smirnov test was initially carried out on the data, and normally distributed measurement data were expressed as $\overline{\mathrm{x}}$ ± standard deviation (SD). Comparisons between two groups were carried out by independent samplet-test. Measurement data that did not conform to the normal distribution were expressed in median. Comparisons between two groups were carried out by Wilcoxon rank sum test, and the test level was α = 0.05. The receiver operating characteristic curves (ROCs) were obtained for each parameter to determine the optimal cutoff value in the differentiation of benign and malignant breast lesions. The area under the ROC curve (AUC) was used to compare the diagnostic capacities of these parameters. The sensitivity and specificity were calculated according to the greatest Youden index (Youden index = sensitivity+ specificity-1). Sensitivity and Specificity are the best when Youden index reach the highest value.

## Results

### Pathological examination results

**Fig. 1:**
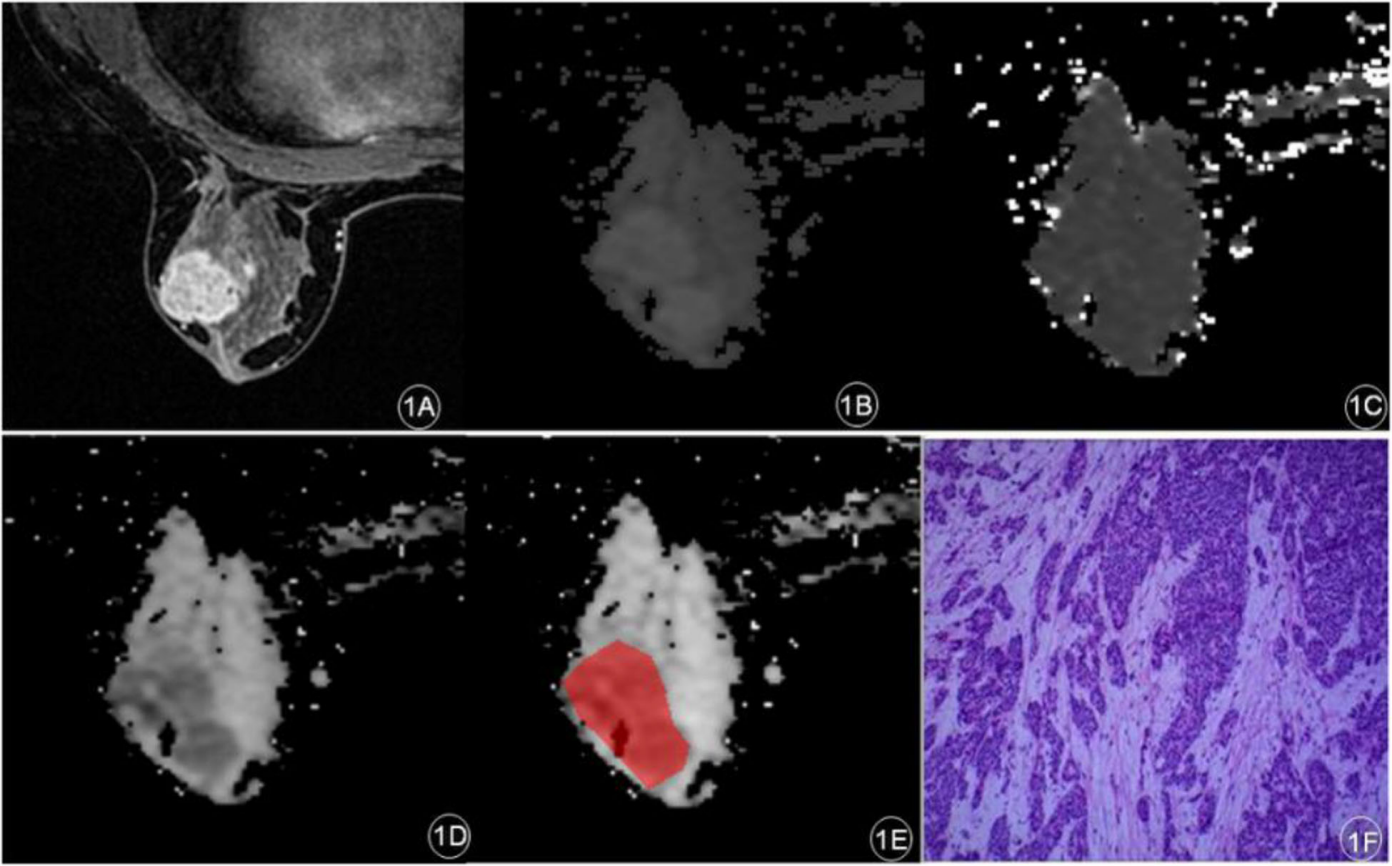
A patient with left breast space occupying pathology and the IVIM parameter map. **a**: Early stage of dynamic enhancement: Left breast mass enhancement with irregular edge; **b**: ADC-slow; **c**: ADC-fast; **d**: **f**; **e**: the region of ROI; **f**: Invasive breast carcinoma (non-special), World Health Organization (WHO) Class II (hematoxylin and eosin [H&E], × 200)

**Fig. 2:**
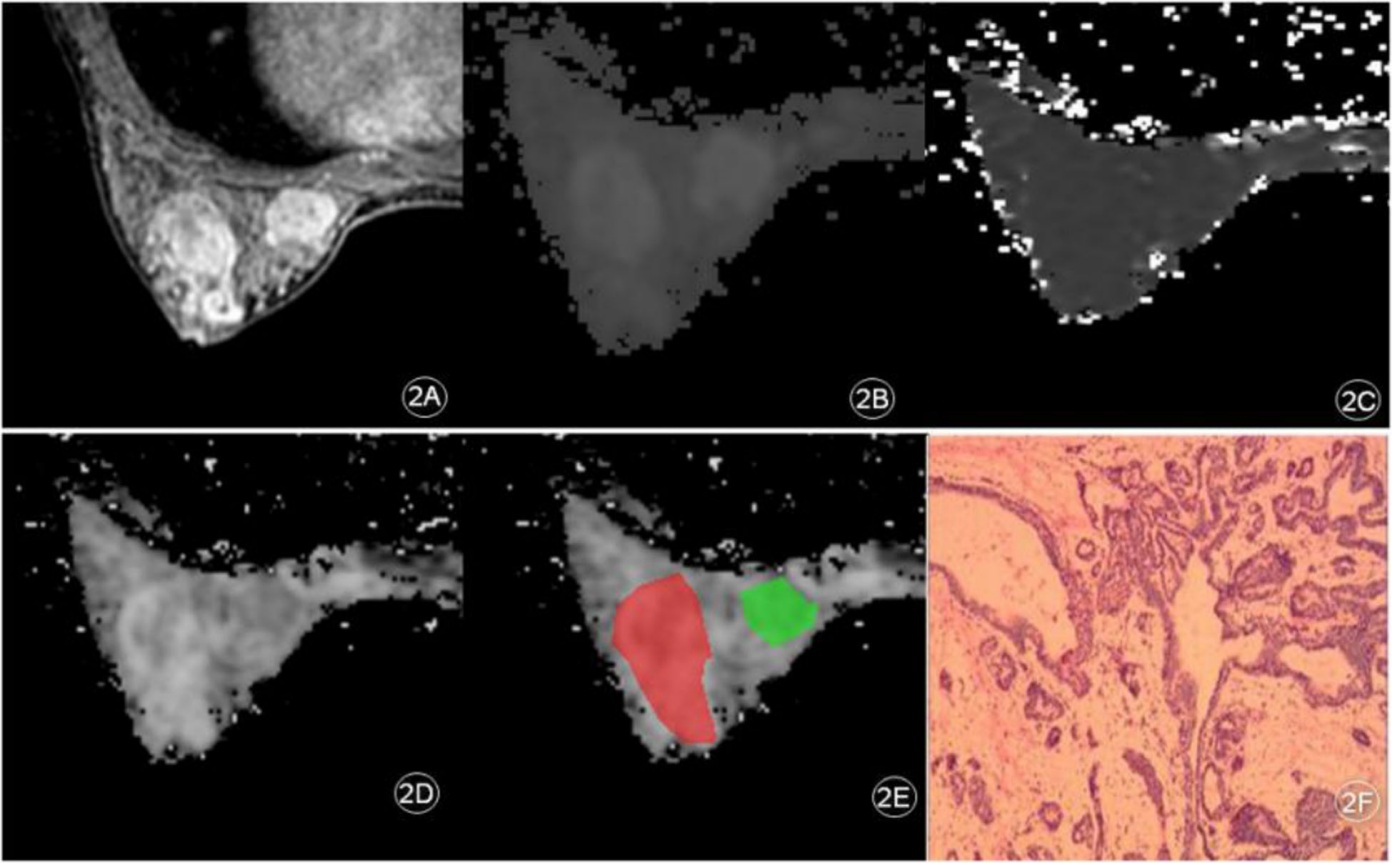
A patient with left breast space occupying pathology and the IVIM Parameter Map. **a**: Early stage of dynamic enhancement: Multiple circular enhancement of the left breast; **b**: ADC-slow; **c**: ADC-fast; **d**: **f**; **e**: the region of ROI; **f**: Left breast disease with ductal dilation (hematoxylin and eosin [H&E], × 200)

In this study, 63 lesions were detected in 55 patients. There were 37 benign lesions in 29 patients, which included 23 fibroadenomas, 6 adenosis, 1 breast cysts, 4 intraductal papillomas, and 3 inflammations of breast. Furthermore, 26 malignant lesions in 26 patients, which included 20 non-specific invasive ductal carcinomas, 5 intraductal carcinomas and 1 patient with squamous cell carcinoma. A case of the non-specific invasive ductal carcinoma is presented in Fig. 1a-f, and a case of the adenosis in combination with duct ectasia is presented in Fig. 2a-f.

### Gray-scale histogram parameter analysis of ADC-slow, ADC-fast and f

The difference in f (minimum, mean, variance, skewness, kurtosis, the 10th percentile and 50th percentile), ADC-fast (kurtosis), ADC-slow (mean and the 50th percentile) was significant (*P* < 0.05) in benign and malignant breast lesions, but the difference in rest parameters was not statistically significant in the benign and malignant breast lesions. As shown in Table 1, the f (minimum, mean, kurtosis, the 10th percentile and 50th percentile) and ADC-slow (mean and the 50th percentile) were significantly lower in malignant breast lesions than in benign lesions, while f (variance, skewness) and ADC-fast (kurtosis) were significantly higher in malignant lesions compared to benign lesions.

**Table 1:** Differences of IVIM histogram parameter in benign and malignant breast lesions

Histogram parameter	Benign lesions(*n* = 37)	Malignant lesions(*n* = 26)	*P* value
f			
minimum	47.0(11.0,89.5)	8.0(−29.3,33.0)	0.004
mean	134.6(111.5166.1)	115.6(97.7126.1)	0.009
variance	767.6(505.51246.3)	1241.0(757.52025.7)	0.032
skewness	−1.2(− 1.7,-0.4)	− 0.5(− 1.0,-0.1)	0.002
kurtosis	0.9(− 0.4,2.7)	−0.2(− 1.0,0.5)	0.002
1th percentile	(1.0,95.0)	(1.0, 58.5)	0.143
10th percentile	105.0(52.0,139.5)	61.0(33.0,93.8)	0.005
50th percentile	137.0(119.5171.5)	122.0(104.8129.3)	0.006
90th percentile	(139.0,192.0)	(140.3169.3)	0.468
99th percentile	(148.5230.0)	(153.8190.3)	0.382
ADC-Fast			
kurtosis	3.8(0.3,8.3)	9.8(2.3,16.0)	0.034
ADC-slow			
Mean	84.2(69.3,98.4)	66.9(60.0,82.8)	0.002
1th percentile	(22.0, 74.0)	(49.0, 75.5)	0.186
10th percentile	(58.5, 80.5)	(58.8, 82.8)	0.189
50th percentile	85.0(69.5,98.0)	66.5(58.9,83.0)	0.000
90th percentile	(72.5, 105.0)	(72.8, 105.3)	0.209
99th percentile	(75.0, 105.0)	(74.8, 111.3)	0.983

**Fig. 3:**
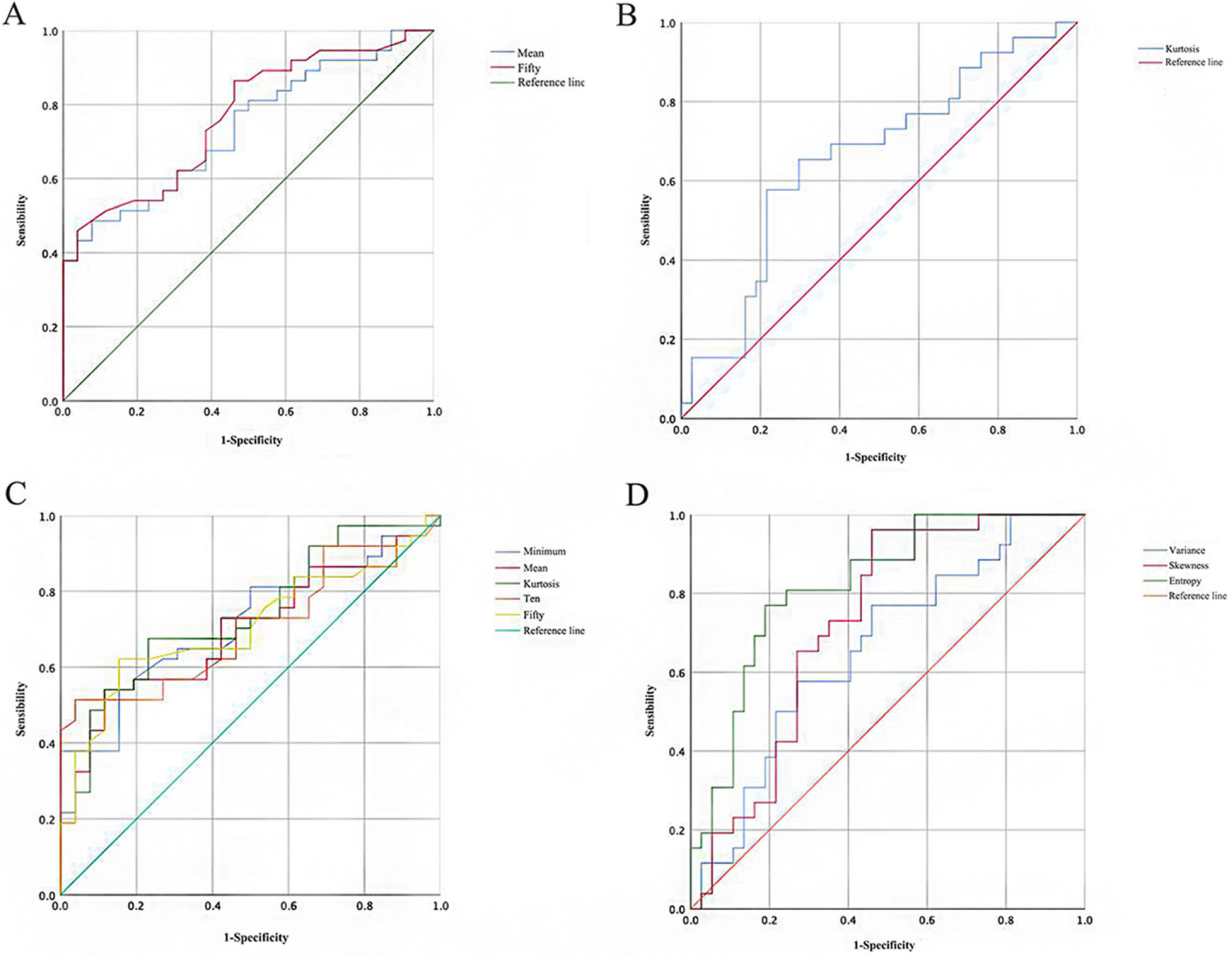
ROC curve of: (**a**) Mean and 50th percentile of ADC-slow. **b** ADC-fast (kurtosis). **c** Minimum, mean, kurtosis, 10th and 50th percentile of f value. **d** Variance, skewness of f value

Table 2 presents the results obtained using the ROC curve to analyze the diagnostic efficacy of the histogram parameters of the IVIM model in benign and malignant breast lesions. According to the ROC of each parameter (Fig. 3), the sensitivity and specificity of each parameter in distinguishing benign and malignant breast lesions were obtained, and the area under the curve (AUC) of each parameter was obtained. The diagnostic efficiency of the 50th percentile of ADC-slow was high. The area under the curve was 0.823 and 0.766, the corresponding sensitivity and specificity was 76.9 and 81.1%, and 45.9 and 96.2%, respectively, and the critical values were 1.78 and 86.5, respectively.

**Table 2:** Efficacy of histogram of IVIM parameters in identifying benign breast lesions

Histogram parameter	AUC	Threshold value	Sensitivity	Specificity
f				
minimum	0.715	43.5	0.541	0.846
mean	0.694	132.5	0.541	0.885
variance	0.660	797.6	0.769	0.541
skewness	0.728	−1.17	0.962	0.541
kurtosis	0.736	0.47	0.676	0.769
10th percentile	0.707	104.5	0.514	0.962
50th percentile	0.705	132.0	0.622	0.846
ADC-Fast				
kurtosis	0.658	9.18	0.577	0.784
ADC-slow				
mean	0.736	85.2	0.486	0.923
50th percentile	0.766	86.5	0.459	0.962

## Discussion

Diffusion-weighted imaging (DWI) can noninvasively detect the brownian motion of water molecules inside and outside cells, which is affected by a variety of factors, including the density of cells, the structure of cell membranes, and macromolecular substances inside and outside cells. In addition, DWI can quantitatively reflect the microenvironment in tissues by measuring the distance that water molecules are allowed to move in a certain period of time. The quantitative index can be expressed through the ADC value, but the ADC value can be easily affected by the perfusion of microcirculatory blood flow in the tissue.

In previous studies, T1WI, T2WI, contrast enhanced T1WI sequence and ADC map have been mostly used for texture analysis. The present study revealed that f (minimum, mean, kurtosis, the 10th percentile and 50th percentile) and ADC-slow (mean and the 50th percentile) in malignant breast lesions were significantly lower than in the benign lesions, while f (variance, skewness) and ADC-fast (kurtosis) in malignant breast lesions were significantly higher than in the benign lesions, indicating that the above parameters can be used for the differential diagnosis of benign and malignant breast lesions due to the different manifestations in benign and malignant breast lesions.

The average gray value of gray histogram reflects the data concentration trend and average level. The higher the gray value means more bright areas in the region of interest. ADC slow represents the true diffusion degree of water molecules in tissues, which can reflect the heterogeneity of tissues. In the present study, the mean ADC-slow and 50th percentile of malignant breast lesions were significantly lower than those of benign breast lesions. The causes were as follows: the cell density of malignant lesions was high and the ratio of the intracellular nucleus to the cytoplasm increased, which led to a decrease in intracellular and extracellular spaces. Furthermore, the heterogeneity of tissues was higher, the limited diffusion of water molecules was more significant [10], and the gray value was lower.

Previous studies have revealed [11] that ADC-fast has limited applications in disease diagnosis due to its poor stability and reproducibility, but it was found that the kurtosis of ADC-fast was significantly different in the benign and malignant lesions in this study. Kurtosis could reflect the peak value of the gray-scale histogram by calculating the distribution of pixels in issues. Furthermore, it is the position of the peak height, which indicates the size of the ADC-fast value under the maximum frequency [12]. In the present study, the kurtosis of ADC-fast in malignant lesions was significantly higher than that in benign lesions, which indicates that the maximum frequency of ADC-fast value in malignant lesions is significantly higher than that in benign lesions. This reflected that the microvascular perfusion area in malignant lesions is significantly higher than that in benign lesions.

In the present study, the histogram parameters of the perfusion fraction f which represented the proportion of the fast diffusion component, include the minimum, mean, variance, skewness, kurtosis, the 10th percentile and the 50th percentile were significantly different between benign and malignant breast lesions. The perfusion fraction f of the tissue represents the proportion of the stroma at different vascular levels to the diseased tissue, which is correlated to the microstructure of the tissue. Tamura et al. [13] considered that the f value of non-invasive breast cancer is significantly higher than that of invasive breast cancer. The present study revealed that the minimum value, mean, kurtosis, the 10th percentile and the 50th percentile of malignant lesions are significantly lower than those of benign breast lesions. By analyzing these reasons, on one hand, the increased neovascularization and solid components of malignant lesions may lead to the compression of microvessels, and the decrease of the signal attenuation ratio is caused by microperfusion; on the other hand, the necrotic cysts of malignant lesions increases, while the rapid diffusion components of the necrotic cysts are almost zero. In the present study, the possible necrotic cystic area was not avoided when selected the ROI. To some extent, it reduceed the f value of malignant lesions.

The variance mainly describes the mean degree of dispersion of the lesion’s characteristic value. The larger the variance was, the more data representing the deviation of the mean value in the lesion, and the higher non-uniformity of the lesion was [14]. The degree of skewness can reflect the asymmetry of the gray-scale signal distribution of the pixel in the particular ROI. The higher the degree is, the higher the complexity of the texture feature in the ROI would be. This indicates an increase in heterogeneity within the lesion [15]. The variance, skewness of the f value of breast malignant lesions in the present study were significantly higher than those of benign lesions, which indicated that the heterogeneity of malignant lesions is significantly higher than that of benign lesions, and was consistent with the pathological characteristics of the lesions.

The present study has some limitations. First, the histology of benign lesions included in the present study was quite different, which reduced the specificity of the differential diagnosis, to a certain extent. As the next step, the investigator will increase the sample size and further study the differences between benign and malignant lesions. Second, the delineation of the ROI is only an analysis of the two-dimensional characteristics of the largest dimension of the lesion, and not an analysis of the texture features of the whole volume of the tumor. Furthermore, the IVIM parameters had many influencing factors. Bokacheva et al. [10] considered that the interval and size of the b value can affect the IVIM parameters, especially on perfusion-related parameters. Therefore, the reasonable selection and optimization of the b value also needs further analysis and research. In addition, proton density, echo time and T2 can also affect these IVIM parameters. These factors would also be controlled as variables in future research, thereby determiming the appropriate diagnostic conditions.

## Conclusion

The histogram analysis of ADC-slow (mean and the 50th percentile), ADC-fast (kurtosis) and f (minimum, mean, kurtosis, the 10th percentile and 50th percentile. Variance, skewness) can provide a more objective and accurate basis for the differential diagnosis of benign and malignant breast lesions.

## Data Availability

The datasets generated and/or analysed during the current study are not publicly available due to the lack of an online platform but are available from the corresponding author on reasonable request.

## Abbreviations

IVIM: Intravoxel incoherent motion
ADC: Apparent diffusion coefficient
MRI: Magnetic resonance imaging
DWI: Diffusion-weighted imaging
T1WI: T1-weighted imaging
T2WI: T2-weighted imaging
SD: Standard deviation
ROC: Receiver operating characteristic
